# Word and pseudoword reading in young adults: an eye-tracking study

**DOI:** 10.1590/2317-1782/20212020333

**Published:** 2022-02-02

**Authors:** Fernanda Marchezini, Peter Maurice Erna Claessens, Maria Teresa Carthery-Goulart

**Affiliations:** 1 Programa de Pós-graduação em Neurociência e Cognição, Universidade Federal de ABC – UFABC – São Bernardo do Campo (SP), Brasil.; 2 Centro de Matemática, Computação e Cognição, Universidade Federal do ABC – UFABC – São Bernardo do Campo (SP), Brasil.; 3 Instituto Nacional de Ciência e Tecnologia sobre Comportamento, Cognição e Ensino – INCT-ECCE – São Carlos (SP), Brasil.; 4 Grupo de Pesquisa em Neurologia Cognitiva e do Comportamento, Departamento de Neurologia, Faculdade de Medicina, Universidade de São Paulo – USP – São Paulo (SP), Brasil.

**Keywords:** Reading, Language, Psycholinguistics, Eye Movements, Eye Movement Measurements, Dyslexia

## Abstract

**Purpose:**

To evaluate and characterize the oculomotor behavior during the reading of words and pseudowords in Brazilian Portuguese organized by frequency, length and regularity and verify its association with performance on neuropsychological tests.

**Methods:**

21 university students, with a mean age of 20.9 years, were submitted to a word and pseudoword reading task (TLPP) from the Anele Battery, in addition to verbal fluency and phonological working memory tests. The patterns of first fixation duration, gaze duration and rate of refixation were studied.

**Results:**

The first fixation duration and the gaze duration were significantly lower for words if compared to pseudowords and the gaze duration was also lower for high-frequency and short words. Significant interactions were also found between verbal fluency performance and the first fixation duration.

**Conclusion:**

Our results demonstrate the applicability of eye tracking to study reading patterns at the word-level in Brazilian Portuguese. The eye tracker can be an additional tool in the investigation of acquired and developmental reading disorders and can assist in the detection of reading difficulties based on comparisons of the oculomotor behavior between fluent and non-fluent readers.

## INTRODUCTION

Decoding can be analyzed by the dual-route model that explains the reading of isolated words through two processes: the lexical route and the phonological route. When a sequence of graphemes is visually processed, the lexical route allows direct access to the word representation in the internal lexicon, being the route used to read familiar regular and irregular words (actually the pronunciation of the latter stimuli cannot be supplied by grapheme-phoneme conversion). The phonological route involves the use of grapheme-phoneme correspondence rules and allows the reading of unfamiliar regular words and pseudowords^(1,2)^. The initial reader uses both reading routes in parallel and becomes consistent in the use of phonological and orthographic clues for the activation or inhibition of words. As he becomes a skilled reader, the reading speed increases, especially in shorter and more frequent words^(3)^.

During reading, the main oculomotor behaviors analyzed are the fixations and the saccades. Fixations are pauses that the eyes make to analyze an area of the word in a region called ‘fovea’, located in the center of the retina and capable of extracting the linguistic information more efficiently^(4,5)^. Saccades are the movements that conduct the eyes from one point to another that will be fixated. The first fixation describes the initial stages of the lexical processing and the total time of all fixations corresponds to the integration of the lexical access with the semantic and morphosyntactic systems, driving to the total word recognition^(6)^ and in skilled readers, the most frequent and shorter words are recognized faster and need less fixations^(7)^. Studies using eye tracking have already been developed with Brazilian young individuals, aiming at characterizing the eye movement patterns during the reading of words and pseudowords. They found that the fixation duration and the number of fixations are higher for pseudowords than for words, for low-frequency words and longer words^(5,8)^, confirming the findings of studies carried out in other languages^(7)^. Regarding the word regularity, significant differences on the oculomotor parameters were found for this psycholinguistic factor in a study conducted in Brazilian Portuguese with young university adults, showing that regular words were processed with lower fixation durations than the irregular ones^(5)^.

Efficient reading also depends on other cognitive functions, such as phonological working memory, a temporary system with limited capacity for storage and processing of verbal information. During reading, it has already been observed through eye tracking techniques that with better phonological working memory capacity, more accurate is the reader’s foveal fixation on the correct position on the word^(9)^ and lower is the occurrence of regressive saccades in order to refixate^(10)^. Another important cognitive function for reading is verbal fluency: the ability to retrieve and evoke the linguistic information stored in semantic memory. The practice of reading strengthens the verbal fluency skill, because it improves metalinguistic awareness and increases vocabulary^(11)^. In fact, the more developed this skill is, the lower the need of rereading and refixating the words^(12)^.

Thinking about the psychological properties and individual cognitive skills that can influence the performance on reading tests, the aim of this study was to analyze the oculomotor behavior during the reading of single words and pseudowords in a population of young adults and confirm the characteristics found in other Brazilian studies. Besides that, by analyzing the oculomotor behavior, we investigated how psycholinguistic factors can interact during reading tasks and how oculomotor parameters are associated with performance on phonological working memory and verbal fluency cognitive tasks. We used the Anele Battery, authorized by the authors, which is a validated protocol for reading assessment which is available for clinical use, in order to demonstrate how eye trackers can complement the reading evaluation.

## METHODS

This study was approved by the Ethics Committee under protocol 15305813.5.0000.0082 - 310.077. Twenty-one right-handed university students, being 8 men and 13 women, with a mean age of 20.9 years, participated in this study and all of them signed an Informed Consent Form. In the initial interview, none of the participants reported visual or hearing deficits uncorrected, attention-deficit / hyperactivity disorder, previous learning difficulties, neurological or psychiatric diseases or using drugs with action on the Central Nervous System. Phonological working memory tests with digit span tasks and verbal fluency tests were also applied. On verbal fluency tests, the participants were asked to generate animal names and words that start with the phonemes /f/, /a/ and /s/, in one minute for each category.

The Stimuli were those of the Word and Pseudoword Reading Task (TLPP)^(13)^ of Anele Battery consisting of 48 words and 24 pseudowords. This task was designed to assess the lexical and phonological routes in reading i.e., lexicality, length, regularity and frequency effects by a balanced distribution of short / long, regular / irregular and high-frequency / low-frequency words in Brazilian Portuguese. To assess the length effect, the TLPP stimuli comprise “short” words (with up to two syllables and no more than 5 letters) and “long” words (three or more syllables or above five letters). For regularity, the Task adopts as “regular” (transparent correspondence between graphemes and phonemes for the pronunciation of the word) and “irregular” when the grapheme-phoneme conversion has ambiguities for pronunciation. Regarding frequency, the elaboration was based on the occurrences of the Portuguese Bank of Brazilian Corpus – PUC-SP^(14)^. In order to manipulate the lexicality effect, the Task included 24 pseudowords that were created from 24 words contained in the battery, keeping the same syllabic structure. The final task resulted in 72 stimuli presented in 12 lists with six stimuli on each one, written in black color on a white background, using the Arial text font, capital letter and size 20. The font size, in pixels, varied depending on the letter, as well as the distance between one letter to another within the same word. There was no variation among the rows, with a distance of 205 pixels among them.

An *Arrington Research* (AR) binocular system equipment with pupil capture was used to record the eye movements at a rate of 60Hz. Information about the oculomotor behavior was extracted from the *ViewPoint* software, version 2.8.6, to interface with the AR equipment. The stimuli were projected on a *Hewlett-Packard* (HP) LCD monitor with resolution of 1440 x 960 pixels with a visible area of 41 x 25.6 cm, positioned at a distance of 40 cm from the chin and forehead support of the participants and the experiments were run in a low-light room. The equipment was calibrated for each participant according to their pupil size and the observers were asked to fix their gaze on 16 points arranged in a grid line and presented in random order on the computer monitor. Before the beginning of each block of words and pseudowords, a fixed point was presented in the center of the screen so that the participants could prepare their eyes to start the next step of the task. All participants were instructed to read the word lists in a low voice and the *ViewPoint* software produced numerical records with the values of the total time of signal capture, the horizontal and vertical positions of fixations for each eye and the horizontal and vertical axes of the pupils. These parameters were used to calculate, offline, the number of fixations, the duration of each fixation and the duration of the first fixation in reading the words. The analysis of the results was executed using *The R Project for Statistical Computing* software (R, version 3.1.2) and the *Jamovi* 1.6.15 software. The significance level adopted was 0.05. Multifactorial ANOVAs were executed to analyze the interaction of fixation durations and psycholinguistic variables among them and with performance on verbal fluency tests and phonological working memory.

During the reading, the oculomotor behavior was analyzed according to these psycholinguistic variables: lexicality, frequency, length and regularity. The dependent variables were: First Fixation Duration (FFD), corresponding to the duration of the first fixation on a word before the first saccade and Gaze Duration (GD), which represents the sum of all fixations within the word, before the saccade to another word. All parameters were analyzed by the mean and by the median of the durations in milliseconds. The medians presented lower values than the means, indicating right skewed distributions and/or presence of excessively long durations, which are violations of normality and it could weaken the assumptions for the analysis of raw data. Therefore, to ensure the validity of approximate normality and the robustness of the measurements, the medians were adopted for all analysis. The probability of refixing each stimulus was also analyzed and defined as *refixation rate* (rate of returns to the stimulus).

## RESULTS

A repeated measures ANOVA revealed main effects of lexicality, length and frequency on First Fixation Duration (FFD) and Gaze Duration (GD), as shown in Table 1, in which the value of these parameters was significantly lower for real and high-frequency words in comparison with the stimuli they were paired with, i.e., pseudowords and low-frequency words. In the isolated effect of length, in particular, we also found that Gaze Duration was significantly lower for short words than for long words, while the First Fixation Duration was higher for short words than for long words. Although, in the effect of length, First Fixation Duration exhibited a *p*-value slightly over 0.05, we observed a large effect size according to the guidelines by Cohen^(15,16)^, with F(1,20) = 4.07; p=0.057; η^2^_p_ = 0.169. No significant differences were found for the isolated effect of regularity.

**Table 1:** Median and standard deviation for First Fixation Duration and Gaze Duration in milliseconds for the main effect of lexicality, frequency and length

			Median	SD	*F*(1,20)	*p*	η^2^_p_
FFD	Lexicality	Words	300	287.41	7.24	0.014	0.266
		Pseudowords	366.66	417.34			
GD	Lexicality	Words	366.66	465.51	46.8	<0.001	0.701
		Pseudowords	700	728.13			
FFD	Frequency	High	300	244.94	5.38	0.031	0.212
		Low	333.33	323.09			
GD	Frequency	High	333.33	399.40	12.4	0.002	0.383
		Low	433.33	431.51			
FFD	Length	Short	333.33	322.16	4.07	0.057	0.169
		Long	300	382.85			
GD	Length	Short	400	503.38	5.77	0.026	0.224
		Long	466.66	595.79			
FFD	Regularity	Regular	300	241.27	0.02	0.875	0.001
		Irregular	300	327.07			
GD	Regularity	Regular	366.66	374.79	0.77	0.390	0.037
		Irregular	366.66	455.75			

Caption: FFD = First Fixation Duration; GD = Gaze Duration; SD = Standard Deviation; *F* = F-Statistic; *p* = significance level*;* η^2^_p_ = partial eta squared - effect size

For Gaze Duration, repeated measures ANOVA showed an interaction effect between lexicality and length (F[1,20] = 16.00; *p* = <0.001, η^2^_p_ = 0.444). Table 2 shows the results of the post-hoc analysis (Tukey), which revealed differences between the psycholinguistic variables, in which GD for short words was lower than for long words, that, in turn, was lower than for short pseudowords and long pseudowords, respectively. A repeated measures ANOVA also revealed a marginally significant three-way interaction effect between frequency, length and regularity, with F(1,20) = 4.57; *p* = 0.045, η^2^_p_ = 0.186, in which, although the obtained p-value is close to the significance level of 0.05, we found a large effect size that could explain the interaction of these three psycholinguistic factors on Gaze Duration. A post-hoc test (Tukey) analyzing all possible interactions revealed that GD in ‘short, high-frequency and regular’ words was significantly lower (median 333.33 ms) than on ‘long, low-frequency and regular’ words (median 466.66 ms), showing that length and frequency are decisive for the difference in time involved in decoding and that ‘long, high-frequency and regular’ words (median 333.33 ms) are read significantly faster than ‘long, low-frequency and regular’ words (median 466.66 ms), showing that frequency, again, can be a decisive factor in reading processing.

**Table 2:** Median and standard deviation for Gaze Duration in milliseconds for the interaction effect between lexicality and length

	Short Words	Long Words	Short Pseudowords	Long Pseudowords	*F*(1,20)	*p*	η^2^_p_
GD	366.66 (409.37)	366.66 (427.31)	566.66 (647.08)	816.66 (792.05)	16.00	<0.001	0.444

Caption: GD = Gaze Duration; *F* = F-Statistic; *p* = significance level; η^2^_p_ = partial eta squared - effect size

In order to evaluate refixation rate, we executed generalized linear mixed model analyses in which we verified the probability of refixations occurring within a word before the observer's gaze leaves the stimulus. This category of linear models extends the general linear model, which considers both continuous and discrete independent variables, either fixed or random across individuals, but with a continuous dependent variable, to binary dependent variables, among other types. In the current study, the act of refixating at least once, or not, was the binary variable. In practice, as is the case in logistic regression, each response was viewed as a realization of a Bernoulli trial, with a certain probability of refixation related to the independent variables through the link function, the logit, or *log*(θ)-*log*(1-θ), a linear function of predictor variables with coefficients estimated through a maximum likelihood method. These analyses were implemented using the Jamovi Software – 2021^(17,18)^ with the Linear Models module^(19)^ and a logistic link function, which is equivalent to mixed logistic regression, but in this case with discrete predictors. The lexicality, length, frequency and regularity characteristics were transformed through contrast coding and represented by a dummy variable with numerical values -0.5 or 0.5. The model is mixed because, while the effects for these variables were considered fixed across the population of individuals, a variable contribution was added per individual as a random factor, with a stipulated normal population distribution. In the current context, this term can be interpreted as representing the interindividual variation in the base rate of refixation for linguistic stimuli in general. We thus seek to analyze the combined psycholinguistic characteristics and study how one variable might influence another in the probability of refixation during the processing of reading. Analyses compared words and pseudowords, including the effect of length and tested the influences of word length, frequency and regularity. In order to check for statistical significance, the Wald test based on a Χ^2^ statistic was adopted, using a chi-squared distribution with one degree of freedom. All analyses considered a random intercept across volunteers, but fixed effects for the experimental manipulations. The effects will be presented through their linear contribution to the logit of the refixation probability, with their corresponding odds serving as effect size measures. The odds factor indicates with which multiplicative constant the probability ratio of refixating versus not refixating, with other variables already taken into account, is modulated by the difference in levels of the independent variable under scrutiny. A positive logit coefficient corresponds to a larger probability of refixation and will correspond to an odds-factor greater than 1.0.

In a first analysis, main and interaction effects between lexicality and length were determined. Short pseudowords were refixated 38.49% of the time, while long pseudowords were refixated 57.14% of the time. Words were refixated 20.95% of the time when short and 31.91% of time when long. The apparent pattern of a larger number of refixations on pseudowords than on words, as well as on the longer stimuli, independently of lexicality, was confirmed in the logistic regression with lexicality and length as factors. The main effects of word-length (long-short) and lexicality (pseudowords-words) were strongly significant, with linear estimates ± standard errors of 0,7308±0,1175 and 1,0512±0,1181, Χ^2^ of 38.67 and 79.19, respectively and *p* < 0.0001 for both. The effect size was large, with odds of 2.077 and 2.861, respectively; the interaction, however, did not reach statistical significance (0.2104±0.2340, Χ^2^ = 0.8081, *p* = 0.37). The odds factor indicating the probability ratio of fixating versus not fixating and was calculated as a part of the mixed logistic model, taking interindividual variation in refixation tendency into account^(20,21)^.

A second mixed logistic regression analysis was conducted with only words in order to investigate the effects of length, regularity and frequency within this group of stimuli. Among the regular words, long and low-frequency ones were refixated 42.18% of the time, the short and low-frequency ones 21.43%, the long and high-frequency ones 20.83% and the short and high-frequency ones 18.37% of the time. Among the irregular words, the rates were 31.55%, 21.77%, 34.69% and 22.02% respectively. The fit of a random intercept model across volunteers indicated statistical significance, according to the Wald chi-squared test, for frequency (low-frequency - high-frequency, linear estimate ± standard error 0.2896±0.1371, Χ^2^ = 4.4634, *p* = 0.035, *odds* 1.336), for length (long-short, 0.6256±0.1375, Χ^2^ = 20.7091, *p* < 0.0001, *odds* 1.869), for the interaction between regularity (irregular-regular) and frequency (-0.7527±0.2743, Χ^2^ = 7.5305, *p* = 0.006, *odds* 0.4711) and marginal significance for the three-way interaction between length, frequency and regularity (-1.0561±0.5482, Χ^2^ = 3.7114, *p* = 0.054, *odds* 0.3478). Positive values for linear estimates and odds greater than 1 indicate a larger number of refixations for long and less frequent words. The main effect of regularity was not significant, and neither were the two-way interactions (all p>0.15); note that a positive value for regularity would mean a larger number for regular than for irregular words. Also, a model with a crossed random intercept individual-item, with similar results in terms of effect size, but in which all *p*-values increased, with loss of significance at 5% level for frequency and for the three-way interaction (although not for the frequency-regularity interaction). Due to the fact that the same effect sizes produced *p*>0.05, one should take a loss of statistical power into account. A paired comparison of conditions with Holm correction shows the main cause of the frequency-regularity interaction to be the difference between high-frequency and low-frequency regular words (p < 0.005; but after correction, p > 0.5 for the same difference among irregular words) and also, to a lesser extent, the difference between irregular and regular words among the high-frequency words (p<0.10). The first interaction modulates the main effect of frequency, and therefore, the effect of frequency seems to be larger for regular words. Complementarily, the marginally significant difference between high-frequency irregular and regular words indicates that the regular words elicit less refixations than would be expected. Although not significant after the Holm correction, the tendency in the difference between high-frequency regular words and low-frequency irregular words is in the same direction as the differences between high-frequency regular words and the other combinations of frequency and regularity. The pattern that emerges is that words that are, at the same time, high-frequency and regular are less often refixated than other words.

Post-hoc analyses of the three-way interaction, that is, between all 28 pairs of combinations of levels of the three independent linguistic variables, showed that the group of ‘long, low-frequency and regular’ words is refixated significantly more often than all the conditions with short words and also more often than the ‘long, high-frequency and regular’ words. ‘Long, high-frequency and irregular’ words are refixated more often than ‘short, high-frequency and regular’ words, and with marginal significance (*p* < 0.10), more than ‘long, high-frequency and regular’ words. It is notable that the refixation rate of this last group is at the level of the short words, in which all the *p*-values for the paired comparison with all of the of the short word conditions is over 0.5 even before the correction, even given the particularly strong main effect of length.

**Figure 1:**
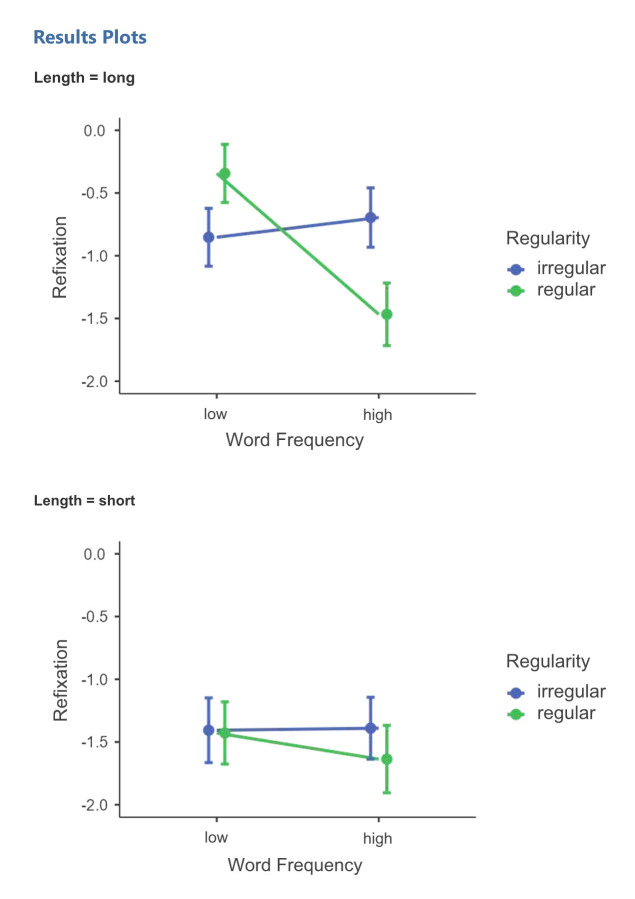
Refixation rate for the three-way interaction effect between length, frequency and regularity

The set of effects (Figure 1) showed that, on average, short words are refixated less often than long words, with the exception of ‘long, high-frequency and regular’ words. Regularity and frequency do not have a meaningful role within the group of short words. The most refixated words are the ‘long, low-frequency and regular’ ones, at a level that is approximately equivalent as that of the long irregular words. Among long-irregular words, frequency does not seem to matter. Among long regular words, however, frequency is decisive in reducing refixation probability from the highest rate (42.28% for low-frequency ones) to the second lowest among the eight word conditions (20.83% for high-frequency ones).

A model with crossed random effects was also used and produced effect sizes compatible with the previously calculated ones, but again with larger numbers for the *p*-values, resulting in the loss of significance at the 5% level, for frequency and for the three-way interaction. Length and interaction between frequency and regularity were maintained as statistically significant effects, which emphasizes the importance of these variables. The frequency x regularity interaction was again caused, in the first place, by the fact that ‘regular and high-frequency’ words elicit less refixations than ‘regular and low-frequency’ words, with a corrected *p*-value <0.10. Post hoc analyses of the three-way interaction also showed, in this case, that the most important difference is between ‘long, low-frequency and regular’ words at the one hand, and short words at the other, especially those that are, additionally, regular and high-frequency. Considering the large number of simultaneous tests (28) involved in the correction and the lower statistical power upon introducing item as a random variable, the difference between long words, regular high-frequency and low-frequency words should be considered statistically significant (corrected p = 0.073).

Finally, as to the neuropsychological tests, the average performance of the sample of participants in the verbal fluency test with semantic categories was 21.52 words per minute, with a standard deviation of 3.47. In the verbal fluency test with phonemic categories, the mean of the three letters was 16.23 words per minute, with standard deviation of 3.23. In order to study the interaction between the dependent variables of eye movement patterns and neuropsychological profile, a split-plot ANOVA and post-hoc analyses showed that individuals with better semantic verbal fluency showed a lower First Fixation Duration (FFD) for long words, with a median of 266.66 ms, when compared to individuals with worse performance on this test, with median of 328.20 ms: F(3,21) = 3.69; *p =* 0.027. No significant interactions between oculomotor patterns and the participants' performance on the phonological working memory were found.

## DISCUSSION

In Table 1, the analysis showed that the First Fixation Duration and the Gaze Duration were lower for words than for pseudowords, since the last ones need grapheme-phoneme conversion and exclusive use of the phonological route, besides they do not give any semantic information to the visual and cognitive systems. In skilled readers, as the population in this research, the saccades move the eyes on some characters between the fixations that will extract the linguistic information from the stimuli^(7,22)^. In these fixations, there is often the processing of neighboring letters that have already been or that will still be run by the saccades while just one letter is fixated on the word^(23)^, having, therefore, a perceptual window of more than one letter in a single fixation^(24)^. The first fixation on the word corresponds to the lexical access, while the total time of all fixations on the word, called *gaze duration*, describes the integration of the lexical access with the semantic system^(5,25)^. As shown in Table 1, we found that short words had a lower Gaze Duration than long words, because they need fewer fixations to be processed, as confirmed by the probability of refixations found in the mixed logistic regression analysis. But we found that short words had longer durations on first fixation than long words. Our explanation for this finding is that for short words, the perceptual window of a single fixation provides access to the orthographic lexicon and allows for semantic integration, resulting in a longer time for its full recognition. On the other hand, for long words, the first fixation is not be enough for lexical access so the reader tends to reduce the time of the first fixation and need more fixations on the word to complete the lexicon access and recognition^(26)^. Our findings replicate and corroborate the findings of previous study with Brazilian adults^(5)^.

Regarding the frequency, we found that high-frequency words had lower First Fixation Duration and Gaze Duration, because the most frequent and predictable words in the language tend to have more familiar orthographic representations with easier and faster recognition^(27)^. Therefore, these words receive a smaller number of fixations and of shorter duration until their full recognition, confirming the results of previous studies carried out in Portuguese and in other languages^(5,7,8)^. The results about the relationship between first fixation duration and gaze duration and psycholinguistic variables, particularly length and frequency effects, were also confirmed by the results found in the mixed logistic regression model, in which we investigated the probability of refixation (or not) of the linguistic stimuli. In this analysis, we found that positive values for linear estimates and *odds* above 1 indicated a greater number of refixations for longer words and for low-frequency words, confirming that high-frequency (and possibly more familiar) and shorter words tend to have a smaller number of fixations^(7,8)^.

Considering the regularity, a previous study of single word reading in adults speakers of Brazilian Portuguese^(5)^ found significant differences between regular and irregular words, so that the latter were processed longer than the former ones (longer first fixation duration and higher number of fixations), suggesting the involvement of a higher cognitive and linguistic load for irregular word reading. Based on the dual-route model, irregular words, which do not use grapheme-phoneme conversion rules, can be activated directly by the lexical route through visual input, so they would take shorter times for full recognition as they do not depend exclusively on the phonological route, which is slower for decoding stimuli. To investigate this finding, we analyzed the regularity effect separately, but we did not find any significant differences in the oculomotor parameters when comparing regular and irregular words. When analyzing the interactions between regularity and other psycholinguistic factors, the *post-hoc* analysis showed that the significant differences were among ‘short, high-frequency and regular’ words, whose gaze duration and refixation rate were lower than ‘long, low-frequency and regular’ words, showing once again that length and frequency are very relevant in the reading process. Another relevant finding was that ‘long, high-frequency and regular’ words had also a significantly lower gaze duration and refixation rate than ‘long, low-frequency and regular’ words, confirming that the frequency can indeed be a determining factor in decoding.

In Figure 1, it is possible to observe that short words are less refixated than long words, but in the analysis with the three psycholinguistic factors together, we find that regularity and frequency are not significant within the short words group, which points to a lower refixation rate for these stimuli anyway. The reduced length allows for the words to be processed with few fixations or even with only one fixation. So in the group of long words, we observed that the most refixated words were the ‘long, low-frequency and regular’, at an approximately similar level than ‘long and irregular’ words, showing that there is no difference related to the frequency. However, among ‘long and regular’ words, the frequency was decisive in reducing the probability of refixation from 42.28% in low-frequency words to 20.83% in high-frequency words. The reason for this reduction can be the nature of the access of irregular words, necessarily carried out through the lexical route, that is, the direct access route from the visual input. In skilled readers, as the sample of the present study, difficulties in spelling or failures are not expected. Therefore, the processing of this group of irregular words would occur directly (as a result of reading from the lexical route). As the sample consists of skilled readers, it was expected that the readers could also read the regular words through the lexical route, considering their efficiency and fluency to decode and recognize words, but even in this population we cannot discard the parallel and simultaneous participation of the phonological route in the reading of regular words. The phonological route is slower and in this additional time of processing, the frequency is decisive in reducing the decoding duration. We must also consider that the stimuli were presented in lists, without any linguistic context, and that the participants may have used the phonological route to reread and confirm what they had already read.

Regarding the oculomotor behavior and its interaction with neuropsychological factors, a study carried out in English^(12)^ using eye-tracking to investigate the reading of sentences with and without semantic ambiguities showed that healthy younger and elder adults, with greater scores in verbal fluency presented longer times in their first reading of the sentence whereas the adults with worse verbal fluency presented the opposite pattern (shorter duration on this parameter). The authors explain that high verbal fluency can justify the longer fixations in first reading of the sentence, being compensated by the lesser need to reread and to refixate sentence segments, while the readers with worse verbal fluency would presented faster first-pass measurements, because they would spend more time reading and rereading all the sentence in order to resolve ambiguities, since they would recruit and organize fewer strategies for word-retrieval. In this study, we found that participants with better semantic verbal fluency had lower First Fixation Duration in long words. Thinking about the interaction with the length, this data may suggest that high verbal fluency may also be associated with less need of rereading. However, differently from that study, the present study investigated single word reading, with no linguistic context that could help the reader to predict or integrate the word’s meaning. The difference of tasks limits the interpretation of our findings so the relationship between verbal fluency and refixation should be investigated in future studies.

We did not find interactions between oculomotor patterns and performance in phonological working memory tests. The type of linguistic stimuli used in this study (single words presented in lists), may have influenced this result and the use of reading protocols with sentences and texts may provide more possibilities for the investigation of associations between reading and working memory.

Finally, considering the studies on this subject in speakers of Brazilian Portuguese, some studies^(5,8)^ have already investigated the correlation of ocular parameters with psycholinguistic variables by using lists of words and pseudowords and found effects of lexicality, frequency, length and regularity that contribute to the discussion about linguistic and cognitive aspects that may be related to reading. In this study, we investigated in more detail the interaction between psycholinguistic variables, ocular parameters and cognitive functions (phonological working memory and verbal fluency). These aspects were less explored in eye tracking and reading studies in Brazilian Portuguese. The measures that we evaluated (first fixation duration, gaze duration and refixation - probability of return to the stimulus) brought additional information to the traditional application of the word and pseudoword reading task that may be relevant for a more accurate clinical diagnosis, as well as helpful to plan interventions for dyslexic patients. It is also important to emphasize that dyslexia can have mild, moderate and severe manifestations. When the difficulty is very mild, for example, in a very early stage of a neurodegenerative condition, a tool that can detect behaviors that suggest an initial dysfunction can be relevant to clinical practice. As a future perspective, more studies with larger and more representative samples are needed, including more variability in education, age, socioeconomic level and also individuals of different regions of Brazil. It will be also necessary to develop tools to facilitate acquisition, analysis and interpretation of eye-tracking data, so that professionals who work in the diagnosis and intervention with individuals with dyslexia can benefit from the additional parameters that can be obtained with this technology. There is a long way to go, and this study aims to inspire further research in this area.

## CONCLUSION

Word and pseudoword reading tests are frequently used for the assessment of people with developmental and acquired dyslexia, informing about the functioning of reading routes. The eye-tracker allows for an objective analysis of the reading process and can be a complementary tool to investigate these aspects, but research is not yet integrated with clinical practice in Brazil. The reason for investigating oculomotor patterns in skilled readers of Portuguese was to contribute to the characterization of the profile of this population. We evidenced the effects of lexicality, frequency and length in stimuli typically used for reading assessment in clinical practice. This study is a proof of concept in which eye-tracking data and reference values obtained in research may have a clinical applicability in the diagnostic investigation. We suggest that eye-tracking data may provide a more sensitive measure of mild deficits. Moreover, some characteristics of oculomotor behavior can be associated with reading performance. More studies are necessary in Portuguese, considering larger samples, different populations, other linguistic stimuli, such as phrases and texts, and investigation of the correlation of oculomotor behavior with higher cognitive functions, especially with phonological working memory and verbal fluency.
