# Supplementary material for: Word and pseudoword reading in young adults: an eye-tracking study
Source: Codas. 2022 Feb 2;34(4):e20200333. doi: 10.1590/2317-1782/20212020333 (PMC9886113; doi:10.1590/2317-1782/20212020333)
Supplement: Translation

# Translation: Leitura de palavras e pseudopalavras em adultos jovens: um estudo de rastreamento ocular

**Authors:** Fernanda Marchezini, Peter Maurice Erna Claessens, Maria Teresa Carthery-Goulart

**Affiliations:** 1 Programa de Pós-graduação em Neurociência e Cognição, Universidade Federal de ABC – UFABC – São Bernardo do Campo (SP), Brasil.; 2 Centro de Matemática, Computação e Cognição, Universidade Federal do ABC – UFABC – São Bernardo do Campo (SP), Brasil.; 3 Instituto Nacional de Ciência e Tecnologia sobre Comportamento, Cognição e Ensino – INCT-ECCE – São Carlos (SP), Brasil.; 4 Grupo de Pesquisa em Neurologia Cognitiva e do Comportamento, Departamento de Neurologia, Faculdade de Medicina, Universidade de São Paulo – USP – São Paulo (SP), Brasil.

**Keywords:** Leitura, Linguagem, Psicolinguística, Movimentos Oculares, Medições dos Movimentos Oculares, Dislexia

## Abstract

**Objetivo:**

Avaliar e caracterizar o comportamento oculomotor durante a leitura de pseudopalavras e palavras do português brasileiro caracterizadas quanto à frequência, extensão e regularidade e verificar sua relação com o desempenho em testes neuropsicológicos.

**Método:**

21 alunos universitários com média de idade de 20,9 anos realizaram a Tarefa de Leitura de Palavras e Pseudopalavras (TLPP) da Bateria Anele, além de testes de fluência verbal e de memória operacional fonológica. Foram estudados os padrões de duração da primeira fixação do olhar no estímulo, duração total das fixações no estímulo e a taxa de refixações (taxa de retornos ao estímulo).

**Resultados:**

A duração da primeira fixação e a duração total das fixações foram significativamente menores para palavras em relação às pseudopalavras, bem como a duração total das fixações foi menor para palavras frequentes e curtas. Também foram encontradas interações significativas entre o desempenho na fluência verbal e a duração da primeira fixação.

**Conclusão:**

Nossos resultados demonstram a aplicabilidade do rastreador ocular para avaliar a leitura no nível da palavra no Português Brasileiro. O rastreador ocular pode ser um instrumento adicional na investigação de transtornos de leitura do desenvolvimento e adquiridos, podendo auxiliar na detecção de dificuldades de leitura a partir da análise de diferenças do comportamento oculomotor entre leitores fluentes e não-fluentes.

## INTRODUÇÃO

A decodificação pode ser analisada pelo modelo teórico de dupla rota que explica a leitura de palavras isoladas através de duas vias: a lexical e a fonológica. A primeira permite que na visualização de uma sequência de grafemas aconteça o acesso direto à representação no léxico mental do leitor, sendo a rota usada para a leitura de palavras conhecidas e palavras irregulares, cuja pronúncia não pode ser aferida pela conversão grafofonêmica. Já a via fonológica envolve a conversão grafema-fonema por meio da aplicação de regras e permite a leitura de palavras não familiares, regulares e pseudopalavras^(1,2)^. À medida que se torna um bom leitor, o indivíduo passa a usar as duas rotas de leitura em paralelo e se torna consistente no uso das pistas fonológicas e ortográficas para a ativação ou inibição de vocábulos, aumentando a velocidade de leitura global, especialmente nas palavras mais curtas e mais frequentes^(3)^.

Os principais comportamentos oculares analisados durante a leitura são as fixações e as sacadas. As fixações são pausas que o olhar faz para analisar na fóvea, região central da retina, uma determinada área da palavra e extrair de forma mais eficaz a informação linguística^(4,5)^. Já as sacadas são os movimentos que os olhos executam de um ponto a outro que deve ser fixado. A primeira fixação na palavra corresponde às etapas iniciais do processamento lexical, já a duração total de todas fixações descreve a integração do acesso lexical com os sistemas semântico e morfossintático, havendo então o total reconhecimento da palavra^(6)^ e nos bons leitores, as palavras mais frequentes e curtas tendem a ser reconhecidas mais rapidamente e precisar de menos fixações^(7)^. Trabalhos usando a técnica de rastreamento ocular já foram realizados no Brasil com o objetivo de caracterizar os padrões oculares durante a leitura de estímulos isolados (palavras e pseudopalavras) em adultos e encontraram que o número e a duração das fixações é maior para pseudopalavras do que para palavras reais, bem como para palavras menos frequentes e mais longas^(5,8)^, confirmando os achados de estudos realizados em outras línguas^(7)^. Ao que se refere à regularidade das palavras, um estudo realizado no Português Brasileiro também com jovens adultos universitários encontrou diferenças significativas dos parâmetros oculomotores nesta condição psicolinguística, sendo as palavras regulares processadas mais rapidamente com tempos de fixações menores que as irregulares^(5)^.

A leitura eficiente também depende de outras funções cognitivas, como a memória operacional fonológica, um sistema de armazenamento e processamento temporário de informações verbais e que tem capacidade limitada. Durante a leitura, já foi observado por meio de técnicas de rastreamento ocular que quanto melhor é a capacidade de memória operacional fonológica do leitor, mais precisa é a fixação da fóvea na posição correta^(9)^ e menor ocorrência de sacadas regressivas no conteúdo linguístico para refixar^(10)^. Outra função cognitiva importante para a leitura é a fluência verbal, habilidade de recuperar e evocar a informação linguística armazenada na memória semântica. A prática da leitura fortalece a habilidade de fluência verbal, pois melhora a consciência metalinguística, aumenta o vocabulário^(11)^ e, quanto melhor for essa habilidade, menor tende ser a necessidade de reler e refixar as palavras^(12)^.

Pensando nas propriedades psicolinguísticas e nas habilidades cognitivas individuais que podem influenciar o desempenho em provas de leitura, o presente estudo teve como objetivo analisar o comportamento oculomotor durante a leitura de palavras e pseudopalavras isoladas em uma população de jovens adultos e confirmar as características encontradas em outros estudos brasileiros. Além disso, através do comportamento oculomotor, buscamos entender neste trabalho como os fatores psicolinguísticos podem interagir durante as tarefas de leitura e como os parâmetros oculomotores podem se correlacionar com o desempenho em tarefas cognitivas de memória operacional fonológica e de fluência verbal. Utilizamos a Bateria Anele com autorização dos autores, que é um protocolo de avaliação de leitura validado e disponível para a prática clínica, a fim de demonstrar como o rastreador ocular pode complementar a avaliação de leitura.

## MÉTODO

Este estudo foi aprovado pelo Comitê de Ética em Pesquisa sob o CAAE no. 15305813.5.0000.0082 e parecer n. 310.077. Participaram do estudo 21 alunos universitários, destros, 8 homens e 13 mulheres, com idade média de 20,9 anos e todos assinaram o Termo de Consentimento Livre e Esclarecido. Na entrevista inicial, nenhum participante relatou ter déficits visuais ou auditivos não corrigidos, Transtorno do Déficit de Atenção e Hiperatividade, dificuldades de aprendizagem prévias, doenças neurológicas ou psiquiátricas ou fazia uso de drogas com ação no Sistema Nervoso Central. Também foram realizados testes de memória operacional fonológica de dígitos e testes de fluência verbal, sendo solicitado que os participantes gerassem nomes de animais e de palavras iniciadas pelos fonemas /f/, /a/ e /s/ em um minuto para cada categoria.

Para avaliar a leitura, foi usada a Tarefa de Leitura de Palavras e Pseudopalavras (TLPP)^(13)^ da Bateria Anele composta por 48 palavras e 24 pseudopalavras. Esta tarefa verifica a participação das rotas lexical e fonológica na leitura, incluindo estímulos com efeitos de lexicalidade, extensão, regularidade e frequência e foi escolhida por ser equilibrada na distribuição de palavras curtas e longas, regulares e irregulares, assim como frequentes e não frequentes na Língua Portuguesa. Quanto à extensão, as autoras da TLPP consideraram “curtas” as palavras com até duas sílabas ou até cinco letras e “longas” as palavras com três ou mais sílabas ou acima de seis fonemas. Para a regularidade, a Tarefa adotou como “regular” toda palavra que corresponde o grafema ao seu fonema com transparência e “irregular” quando a correspondência grafofonêmica não segue uma regra transparente na conversão. Ao que se refere à frequência, a elaboração foi baseada nas ocorrências do Banco de Português do *Corpus* Brasileiro da PUC-SP^(14)^. Para manipular o efeito de lexicalidade, a Tarefa incluiu 24 pseudopalavras que foram criadas a partir de 24 palavras reais da bateria, mas que tiveram suas letras e/ou sílabas trocadas ou omitidas, porém com a mesma estrutura silábica. A tarefa final totalizou 72 estímulos apresentados em 12 listas com seis estímulos em cada uma, escritos em fonte Arial, caixa alta, cor preta, tamanho 20, justificados e em fundo branco. O tamanho da letra, em *pixels*, variava discretamente a depender da letra, assim como a distância entre uma letra e outra dentro de uma mesma palavra. Não houve variação entre as linhas, com distância de 205 *pixels* entre elas.

Para registro dos movimentos oculares foi usado o equipamento *Arrington Research* (AR) de sistema binocular com captura das pupilas a uma taxa de 60Hz. As informações do comportamento oculomotor foram extraídas do *software ViewPoint*, versão 2.8.6, à interface com o equipamento AR. Os estímulos foram projetados em um monitor LCD *Hewlett-Packard* (HP) em resolução de 1440 x 960 pixels com área visível de 41 x 25,6 cm, posicionado a uma distância de 40 cm do apoio de queixo e testa dos participantes e os experimentos foram executados em uma sala com baixa iluminação. O equipamento foi calibrado para cada participante em função do tamanho da sua pupila e foi solicitado ao participante fixar o olhar em 16 pontos dispostos em linha de grade e apresentados em ordem aleatória sobre o monitor do computador. Antes do início de cada bloco de palavras ou pseudopalavras foi apresentado um ponto fixo no centro da tela para que o participante preparasse o olhar para iniciar a próxima etapa da tarefa. Todos foram instruídos a ler mentalmente as listas de palavras e o *software ViewPoint* produziu registros numéricos com os valores de tempo total da captura de sinais, as posições horizontais e verticais das fixações para cada olho e os eixos horizontais e verticais das pupilas. Esses parâmetros foram usados para calcular, *off-line*, o número de fixações, a duração de cada fixação e a duração da primeira fixação na leitura das palavras. A análise dos resultados foi realizada pelo *software The R Project for Statistical Computing* (R, versão 3.1.2) e pelo *software Jamovi* 1.6.15 e o nível de significância adotado foi de 0,05. Foram realizadas ANOVAs multifatoriais para as análises de interação das durações das fixações e das variáveis psicolinguísticas entre si e com o desempenho nos testes de fluência verbal e de memória operacional fonológica.

O comportamento oculomotor durante a leitura silenciosa foi analisado em função das seguintes variáveis psicolinguísticas: lexicalidade, frequência, extensão e regularidade. As variáveis dependentes foram: Duração da Primeira Fixação (DPF), correspondente à duração do primeiro olhar na palavra antes da primeira sacada e Duração Total das Fixações (DTF), correspondente à soma de todas as fixações realizadas dentro da palavra antes do olhar executar a sacada para o próximo estímulo. Todos os parâmetros foram analisados pela média e pela mediana da duração em milissegundos. As medianas apresentaram valores menores que as médias, indicando distribuições positivamente assimétricas e/ou presença de durações excessivamente longas, que são violações de normalidade e que prejudicariam os pressupostos para as análises dos dados brutos. Portanto, para garantir a validade da normalidade aproximativa e a robustez das medidas, foram adotadas as medianas para todas as análises. Também foi analisada a probabilidade de refixar cada estímulo, aqui definida como taxa de refixações (taxa de retornos ao estímulo).

## RESULTADOS

ANOVA de medidas repetidas revelou na Duração da Primeira Fixação (DPF) e na Duração Total das Fixações (DTF) o efeito principal de lexicalidade, extensão e de frequência conforme descrito na Tabela 1, onde o valor desses parâmetros foi significativamente menor para palavras reais e palavras frequentes em comparação com seus pares: pseudopalavras e palavras infrequentes. Particularmente no efeito isolado de extensão, encontramos também que a Duração Total das Fixações foi significativamente menor para palavras curtas que para palavras longas, enquanto a Duração da Primeira Fixação foi maior para palavras curtas que para palavras longas. Embora a Duração da Primeira Fixação no efeito de extensão tenha apresentado *p* valor ligeiramente maior do que 0,05, observamos tamanho de efeito grande de acordo com o guia de interpretação de Cohen^(15,16)^, com F(1,20) = 4,07; *p* = 0,057, η^2^_p_ = 0,169. Não foram encontradas diferenças significativas para o efeito isolado de regularidade.

**Tabela 1:** Valores de mediana e desvio-padrão para a Duração da Primeira Fixação e Duração Total das Fixações em milissegundos em função do efeito principal de lexicalidade, de frequência e de extensão

			Mediana	DP	*F*(1,20)	*p*	η^2^_p_
DPF	Lexicalidade	Palavras	300	287,41	7,24	0,014	0,266
		Pseudopalavras	366,66	417,34			
DTF	Lexicalidade	Palavras	366,66	465,51	46,8	<0,001	0,701
		Pseudopalavras	700	728,13			
DPF	Frequência	Alta	300	244,94	5,38	0,031	0,212
		Baixa	333,33	323,09			
DTF	Frequência	Alta	333,33	399,40	12,4	0,002	0,383
		Baixa	433,33	431,51			
DPF	Extensão	Curta	333,33	322,16	4,07	0,057	0,169
		Longa	300	382,85			
DTF	Extensão	Curta	400	503,38	5,77	0,026	0,224
		Longa	466,66	595,79			
DPF	Regularidade	Regular	300	241,27	0,02	0,875	0,001
		Irregular	300	327,07			
DTF	Regularidade	Regular	366,66	374,79	0,77	0,390	0,037
		Irregular	366,66	455,75			

Legenda: DPF = Duração da Primeira Fixação; DTF = Duração Total das Fixações; DP = Desvio Padrão; *F* = estatística F do Teste ANOVA; *p* = nível de significância*;* η^2^_p_ = eta-quadrado parcial - tamanho do efeito

Para a Duração Total das Fixações, ANOVA de medidas repetidas mostrou efeito de interação entre lexicalidade-extensão (F[1,20] = 16,00; *p* = <0,001, η^2^_p_ = 0,444). A Tabela 2 mostra os resultados da análise *post-hoc* (*Tukey*) que revelou diferenças entre as variáveis psicolinguísticas, onde a DTF em palavras curtas foi menor que em palavras longas, que por sua vez, foi menor que em pseudopalavras curtas e pseudopalavras longas, respectivamente. ANOVA de medidas repetidas também revelou efeito de interação tripla marginalmente significativo entre frequência-extensão-regularidade, com F(1,20) = 4,57; *p* = 0,045, η^2^_p_ = 0,186, onde embora o *p*-valor obtido tenha sido próximo ao nível de significância de 0,05, encontramos um tamanho de efeito grande que poderia justificar a interação desses três fatores psicolinguísticos na Duração Total das Fixações. O teste *post-hoc* (*Tukey*) que analisou todas as interações possíveis revelou que a DTF em palavras curtas-frequentes-regulares foi significativamente menor (mediana 333,33 ms) que nas palavras longas-infrequentes-regulares (mediana 466,66 ms), mostrando que extensão e frequência são determinantes para a diferença no tempo envolvido na decodificação, e que palavras longas-frequentes-regulares (mediana 333,33 ms) são lidas significativamente mais rápido que palavras longas-infrequentes-regulares (mediana 466,66 ms), mostrando que a frequência mais uma vez pode ser fator determinante para o processamento da leitura.

**Tabela 2:** Valores de mediana e desvio-padrão para a Duração Total das Fixações em milissegundos em função do efeito de interação entre lexicalidade-extensão

	Palavras Curtas	Palavras Longas	Pseudopalavras Curtas	Pseudopalavras Longas	*F*(1,20)	*p*	η^2^_p_
DTF	366,66 (409,37)	366,66 (427,31)	566,66 (647,08)	816,66 (792,05)	16,00	<0,001	0,444

Legenda: DTF = Duração Total das Fixações; *F* = estatística F do Teste ANOVA; *p* = nível de significância*;* η^2^_p_ = eta-quadrado parcial - tamanho do efeito

Para avaliar a taxa de refixações, fizemos análises de modelos lineares generalizados mistos nas quais verificamos a probabilidade de refixações acontecerem dentro de uma palavra antes do olhar do participante deixar o estímulo. Essa categoria de modelos lineares estende o modelo linear geral, que contempla variáveis independentes contínuas e discretas, fixas ou randômicas sobre indivíduos, mas agora com uma variável dependente contínua para variáveis dependentes binárias, entre outras. Neste estudo, o ato de refixar pelo menos uma vez, ou não, foi a variável binária. Na prática, como acontece em regressão logística, cada resposta foi vista como a realização de uma tentativa-*Bernoulli* com uma certa probabilidade de refixação relacionada às variáveis independentes pela função-*link*, o *logit*, ou *log*(θ)-*log*(1-θ). A função-*link* é uma função linear das variáveis preditoras com coeficientes a serem estimados por um método de máxima verossimilhança. Estas análises foram implementadas no *software Jamovi* - 2021^(17,18)^ com o módulo *Linear Models* ^(19)^ e uma função-*link* logística, o que equivale à regressão logística mista mas, neste caso, com preditores discretos. As características de lexicalidade, extensão, frequência e regularidade foram transformadas através de codificação de contraste e representadas por um preditor *dummy* com valor numérico -0,5 ou 0,5. O modelo é misto porque enquanto os valores de efeito para estas variáveis foram considerados fixos sobre a população de indivíduos, foi acrescentado um termo variável por indivíduo, como um fator aleatório com distribuição normal estipulada sobre a população. No contexto atual, este termo pode ser interpretado como representando a variação interindividual na taxa-base da refixação de estímulos linguísticos em geral. Buscamos, assim, analisar as características psicolinguísticas conforme a sua combinação e verificar como uma variável pode influenciar a outra na probabilidade de refixar palavras durante o processamento da leitura. Foram realizadas as análises para comparar palavras com pseudopalavras, incluindo o efeito de extensão e para verificar os papeis da extensão, frequência e regularidade das palavras. Foi adotado o teste de *Wald*, com estatística Χ^2^, para aferir significância estatística, usando uma distribuição qui-quadrado e sempre com um grau de liberdade. Todas as análises foram de intercepto randômico sobre os voluntários, mas de efeitos fixos para as manipulações experimentais. Os efeitos serão apresentados através da contribuição linear ao *logit* da probabilidade de refixar, com os *odds* correspondentes como tamanho de efeito. O fator *odds* indica com qual constante multiplicativa a razão da probabilidade de refixar *versus* a de não refixar, já considerando as outras variáveis, é modulada pela diferença em níveis da variável independente em questão. Um coeficiente para o *logit* positivo corresponde a uma maior probabilidade de refixação e corresponderá a um fator-*odds* maior que 1.

Em uma primeira análise, foram determinados os efeitos principais e a interação entre lexicalidade e extensão. Pseudopalavras curtas foram refixadas 38,49% das vezes, enquanto pseudopalavras longas foram refixadas 57,14% das vezes. Palavras foram refixadas 20,95% das vezes quando curtas e 31,91% das vezes quando longas. O aparente padrão de maior número de refixações nas pseudopalavras do que nas palavras, bem como nos estímulos longos independente da lexicalidade, foi confirmado na regressão logística com lexicalidade e extensão como fatores. Os efeitos principais de extensão (longas-curtas) e lexicalidade (pseudopalavras-palavras) foram fortemente significativos, com estimativa linear ± erro padrão de 0,7308±0,1175 e 1,0512±0,1181, Χ^2^ de 38,67 e 79,19, respectivamente e, *p* < 0,0001 para ambos. O tamanho de efeito foi grande, com *odds* de 2,077 e 2,861, respectivamente, enquanto a interação não alcançou significância estatística (0,2104±0,2340, Χ^2^ = 0,8081, *p* = 0,37). O fator *odds* foi calculado como parte do modelo logístico misto, levando em conta a variação interindividual na tendência de realizar as refixações^(20,21)^.

Uma segunda análise de regressão logística mista foi realizada somente com as palavras para investigar os efeitos de extensão, regularidade e frequência dentro deste grupo de estímulos. Entre as palavras regulares, as que eram longas e infrequentes foram refixadas 42,18% das vezes, as curtas e infrequentes 21,43%, as longas e frequentes 20,83% e as curtas e frequentes 18,37% das vezes. Entre as palavras irregulares, as taxas foram de 31,55%, 21,77%, 34,69%, e 22,02% respectivamente. O ajuste do modelo com intercepto randômico para os voluntários indicou significância estatística, conforme o teste chi-quadrado de *Wald*, para frequência (infrequentes-frequentes, estimativa linear ± erro padrão 0,2896±0,1371, Χ^2^ = 4,4634, *p* = 0,035, *odds* 1,336), para extensão (longas-curtas, 0,6256±0,1375, Χ^2^ = 20,7091, *p* < 0,0001, *odds* 1,869), para a interação entre regularidade (irregular-regular) e frequência (-0,7527±0,2743, Χ^2^ = 7,5305, *p* = 0,006, *odds* 0,4711) e significância marginal para a interação tripla entre extensão, frequência e regularidade (-1,0561±0,5482, Χ^2^ = 3,7114, *p* = 0,054, *odds* 0,3478). Valores positivos para as estimativas lineares e *odds* acima de 1 indicam maior número de refixações para palavras mais longas e menos frequentes. O efeito principal de regularidade não foi significativo, bem como para as outras interações duplas (todos com *p* > 0,15), no entanto um valor positivo para regularidade significaria maior número de refixações para palavras regulares em comparação com irregulares. Também foi ajustado um modelo com intercepto randômico indivíduo e item cruzado, com resultados parecidos em termos de tamanho de efeito, mas no qual todos os *p*-valores subiram, perdendo a significância no nível de 5% para a frequência e para a interação tripla (mas não para a interação frequência e regularidade). Pelo fato que os mesmos tamanhos de efeito produziram *p* > 0,05, deve-se levar em conta a perda do poder estatístico. Uma comparação de condições por pares com correção de *Holm* mostra que a principal causa da interação frequência *x* regularidade é a diferença entre palavras regulares frequentes e raras (*p* < 0,005; mas após correção, *p* > 0,5 entre palavras irregulares para a mesma diferença) e também, em menor grau, a diferença entre palavras irregulares e regulares em palavras frequentes (*p* < 0,10). A primeira interação modula o efeito principal de frequência, portanto, o efeito de frequência parece ser maior para palavras regulares. De forma complementar, a diferença marginalmente significativa entre palavras frequentes irregulares e regulares indica que as regulares provocam menos refixações do que seria esperado. Apesar de não significativa após correção de *Holm*, a tendência de diferença entre palavras regulares frequentes e palavra irregulares infrequentes está no mesmo sentido que as diferenças entre palavras regulares frequentes e as outras combinações de frequência e regularidade. O padrão que surge é que palavras ao mesmo tempo frequentes e regulares são refixadas menos vezes que outras palavras.

Análises *post-hoc* da interação tripla, isto é, entre todos os 28 pares de combinações dos níveis das três variáveis linguísticas independentes, mostraram que o grupo de palavras longas-infrequentes-regulares é refixado significativamente mais que todas as condições com palavras curtas e também mais que palavras longas-frequentes-regulares. Palavras longas-frequentes-irregulares são refixadas mais vezes que curtas-frequentes-regulares e com significância marginal (*p* < 0,10), mais do que palavras longas-frequentes-regulares. Chama a atenção que a taxa de refixação deste último grupo é no nível das palavras curtas, onde todos os *p*-valores para a comparação pareada com cada uma das condições de palavras curtas ficaram acima de 0,5 mesmo antes da correção, apesar do efeito principal particularmente robusto de extensão.

O conjunto de efeitos (Figura 1) mostrou que, em média, as palavras curtas são menos refixadas que as palavras longas, com a exceção de palavras longas-frequentes-regulares. Regularidade e frequência não cumprem papel significativo dentro do grupo de palavras curtas. As palavras mais refixadas são as longas-infrequentes-regulares, em nível aproximadamente equivalente a palavras longas-irregulares. Entre palavras longas-irregulares, a frequência não parece importar. Entre palavras longas-regulares, no entanto, a frequência é determinante em reduzir a probabilidade de refixação da maior taxa (42,28% para infrequentes) para a segunda menor entre as oito condições com palavras (20,83% para frequentes).

**Figura 1:** Taxa de refixações para a interação tripla entre extensão, frequência e regularidade

Legenda: SE = Standard Error

Um modelo com efeitos randômicos cruzados também foi usado e produziu tamanhos de efeito compatíveis com os calculados anteriormente, mas novamente com valores maiores para todos os *p*-valores, resultando a perda de significância, em nível de 5%, para frequência e para a interação tripla. Extensão e interação entre frequência e regularidade foram mantidas como efeitos estatisticamente significativos, destacando a importância destas variáveis. A interação frequência *x* regularidade foi novamente motivada, em primeiro lugar, pelo fato que palavras regulares frequentes provocam menos refixações que palavras regulares infrequentes, com *p* corrigido < 0,10. As análises post-hoc da interação tripla também mostraram, neste caso, que a diferença mais importante está entre palavras longas-infrequentes-regulares, e palavras curtas, especialmente aquelas que adicionalmente são regulares e frequentes. Considerando o grande número de testes simultâneos (28) envolvidos na correção e o menor poder estatístico ao introduzir item como variável aleatória, a diferença entre palavras longas, regulares frequentes e infrequentes deve ser considerada estatisticamente significativa (*p* corrigido = 0,073).

Por fim, em relação aos testes neuropsicológicos, o desempenho médio da amostra de participantes no teste de fluência verbal por categoria semântica foi de 21,52 palavras por minuto, com desvio-padrão de 3,47. Para o teste de fluência verbal por categoria fonêmica, a média das três letras foi de 16,23 palavras por minuto, com desvio-padrão de 3,23. Para estudar a interação entre as variáveis dependentes dos padrões oculares e o perfil neuropsicológico, ANOVA *split-plot* e análise *post-hoc* mostraram que os indivíduos com melhor fluência Verbal Semântica apresentaram menor duração da primeira fixação (DFT) em palavras longas, com mediana de 266,66 ms, em relação aos indivíduos com desempenho pior nesse teste, com mediana de 328,20 ms: F(3,21) = 3,69; *p =* 0,027. Não foram encontradas interações significativas entre os padrões oculomotores e o desempenho dos participantes nas provas de memória operacional fonológica.

## DISCUSSÃO

As análises na Tabela 1 mostraram que a Duração da Primeira Fixação e a Duração Total das Fixações foram menores para palavras do que para as pseudopalavras, uma vez que estas últimas requerem conversão grafema-fonema e uso exclusivo da rota fonológica, além de não fornecerem nenhuma informação semântica aos sistemas visual e cognitivo. Em bons leitores, como é o caso da população estudada neste trabalho, as sacadas percorrem alguns caracteres entre as fixações que extraem a informação linguística dos estímulos^(7,22)^ e frequentemente, nestas fixações, acontece o processamento de letras vizinhas que já foram ou que serão ainda percorridas pelas sacadas enquanto apenas uma letra é fixada na palavra^(23)^, havendo portanto, uma janela perceptual de mais de uma letra em uma única fixação^(24)^. A primeira fixação na palavra corresponde ao acesso lexical, já a duração total de todas fixações na palavra descreve a integração desse acesso com o sistema semântico^(5,25)^. Conforme descrito na Tabela 1, encontramos que palavras curtas apresentaram Duração Total das Fixações menor que palavras longas, pois necessitaram de um menor número de fixações para serem processadas, como confirmado pela análise da probabilidade de refixações, realizada através da regressão logística mista. Mas encontramos que as palavras curtas apresentaram durações de Primeira Fixação maiores que palavras longas, o que pode ser explicado pelo fato de que nas palavras curtas a janela perceptual de uma única fixação pode dar conta de fazer o acesso ao léxico ortográfico e sua integração semântica, levando mais tempo para o seu reconhecimento completo. Já nas palavras longas, a primeira fixação não seria suficiente para o acesso ao léxico, fazendo o leitor diminuir o tempo desta primeira fixação e precisar de mais fixações dentro da palavra para completar o seu acesso lexical e reconhecimento^(26)^, confirmando os achados de outro estudo brasileiro^(5)^.

Em relação à frequência, encontramos neste estudo que palavras frequentes apresentaram menor Duração da Primeira Fixação e do Total das Fixações, pois estímulos mais frequentes ao leitor e mais previsíveis na língua tendem a ter representações ortográficas mais familiares e podem ter seu reconhecimento mais fácil e rápido^(27)^. Sendo assim, recebem menor número de fixações e em menor duração até seu completo reconhecimento, confirmando os achados também de outros estudos realizados em Português e em outras línguas^(5,7,8)^. Os resultados relacionados às durações da primeira e do total das fixações nos fatores de extensão e frequência também foram confirmados pelos resultados encontrados no modelo de regressão logística mista, onde buscamos verificar a probabilidade dos estímulos linguísticos serem ou não refixados. Nesta análise, encontramos que valores positivos para as estimativas lineares e *odds* acima de 1 indicaram maior número de refixações para palavras mais longas e para palavras menos frequentes, confirmando que palavras mais frequentes (e possivelmente mais familiares) e menos extensas tendem a apresentar menor número de fixações^(7,8)^.

Considerando o fator de regularidade, o estudo brasileiro realizado anteriormente com leitura de palavras isoladas^(5)^ em adultos encontrou diferenças significativas entre palavras regulares e irregulares, tendo sido as últimas mais demoradas que as primeiras tanto nos parâmetros de primeira fixação como no total das fixações, sugerindo envolvimento de maior carga cognitiva e linguística na decodificação de palavras irregulares. Tomando como base o modelo teórico de dupla-rota, as palavras irregulares, que não seguem regras de conversão grafofonêmica, poderiam ser ativadas diretamente pela rota lexical através do *input* visual, podendo assim levar menos tempo para o seu reconhecimento completo por não demandarem exclusivamente da rota fonológica, que é mais lenta na decodificação. Deste modo, buscamos investigar a influência da regularidade nas palavras nos processos de leitura, porém isoladamente não encontramos neste estudo nenhuma diferença significativa nos parâmetros oculomotores na comparação de palavras regulares com irregulares. Então, ao analisarmos a regularidade interagindo com os outros fatores psicolinguísticos, encontramos diferenças a serem consideradas, porém as análises *post-hoc* mostraram que as diferenças altamente significativas estavam entre as palavras curtas-frequentes-regulares, que tiveram sua Duração Total de Fixações e taxa de refixação menores que palavras longas-infrequentes-regulares, mostrando mais uma vez que extensão e frequência cumprem papel importante no processamento da leitura. Outro achado relevante foi encontrado em palavras longas-frequentes-regulares, que tiveram seus tempos totais de fixação e taxa de refixação também significativamente menores que palavras longas-infrequentes-regulares, confirmando que a frequência de fato pode ser fator determinante na decodificação.

Na Figura 1, pode-se observar que, de forma geral, palavras curtas são menos refixadas que palavras longas, mas na análise dos três fatores psicolinguísticos juntos, verificamos que a regularidade e a frequência não cumprem papel significativo dentro do grupo de palavras curtas, sendo estas pouco refixadas de qualquer maneira, possivelmente pela extensão curta ser fator suficiente para que as palavras sejam já processadas com poucas fixações ou, por vezes, apenas uma fixação. Pensando, então, no grupo de palavras longas, observamos que as mais refixadas foram as longas-infrequentes-regulares, em nível aproximadamente equivalente a palavras longas-irregulares, onde nestas últimas a frequência mostrou não fazer diferença. Entre palavras longas-regulares, no entanto, a frequência foi determinante em reduzir a probabilidade de refixação de 42,28% nas infrequentes para 20,83% nas frequentes. A explicação para esse achado pode estar na natureza do acesso de palavras irregulares, realizado necessariamente pela rota lexical, a via de acesso direto pelo *input* visual. Em bons leitores, como é o caso da amostra deste estudo, não são esperadas dificuldades ou falhas na representação/memória ortográfica e o processamento desse grupo de palavras irregulares aconteceria de forma direta. Sendo a mostra composta por bons leitores, também era esperado que esses participantes lessem as palavras regulares pela via lexical, considerando sua eficiência e fluência na decodificação e no reconhecimento de palavras, porém mesmo nesta população não podemos descartar a participação paralela e simultânea da rota fonológica na leitura de palavras regulares. A via fonológica é mais lenta e, neste tempo adicional de processamento, a frequência mostra-se, então, determinante em reduzir a duração da decodificação. Também não se pode desconsiderar o fato dos estímulos terem sido apresentados em listas, sem nenhum contexto linguístico, e os participantes terem feito uso da via fonológica para reler e confirmar o que já haviam lido anteriormente.

Em relação ao comportamento oculomotor e sua interação com fatores neuropsicológicos, um estudo realizado na língua inglesa^(12)^ usando rastreamento ocular para investigar a leitura de sentenças com e sem ambiguidades semânticas mostrou que adultos saudáveis, tanto jovens como idosos, com maior fluência verbal apresentaram maiores durações das fixações na primeira passagem do olhar pelas frases e que adultos com pior fluência verbal apresentaram o inverso, isto é, menor duração neste parâmetro. Os autores explicam que a alta fluência verbal pode justificar as fixações da primeira leitura da oração serem mais demoradas e sendo compensadas pela menor necessidade de reler e refixar segmentos da sentença, ao passo que leitores com fluência verbal pior apresentariam medidas de primeira passagem mais rápidas pois gastariam mais tempo lendo e relendo toda a sentença posteriormente a fim de resolver ambiguidades, já que recrutariam e organizariam menos estratégias para o resgate de palavras. No presente estudo, encontramos que os participantes com melhor fluência verbal semântica apresentaram menor Duração da Primeira Fixação em palavras longas. Pensando na interação com o fator de extensão, esse dado pode sugerir que a alta fluência verbal possa também estar associada à redução da necessidade de reler, porém aqui, diversamente do estudo citado, a leitura foi realizada através de palavras isoladas, sem nenhum contexto linguístico que ajudasse o leitor na previsibilidade das palavras ou na integração com o significado, algo que deve ser considerado para a limitação da interpretação deste resultado e deve ser melhor investigado em estudos futuros.

Não encontramos interações entre os padrões oculomotores e o desempenho nas provas de memória operacional fonológica. O tipo de estímulo linguístico usado neste estudo, que foi de palavras isoladas apresentadas em listas, pode ter influenciado neste resultado e o uso de protocolos de leitura com frases ou textos pode fornecer mais possibilidades para a investigação de correlações entre a leitura e a função da memória operacional.

Por fim, tendo em vista a produção nacional neste tema, estudos brasileiros^(5,8)^ já buscaram investigar a correlação dos parâmetros oculares com variáveis psicolinguísticas através do uso de listas de palavras e pseudopalavras, encontrando efeitos isolados de lexicalidade, frequência, extensão e regularidade que contribuem para a discussão de alguns aspectos linguísticos e cognitivos que podem estar envolvidos no processamento da leitura. Neste estudo, buscamos investigar mais detalhadamente a relação existente na interação entre as variáveis psicolinguísticas e dos parâmetros oculares com algumas funções cognitivas, como a memória operacional fonológica e a fluência verbal, aspectos ainda pouco abordados nos estudos de rastreamento ocular e leitura no Português Brasileiro. As medidas que avaliamos (latência para a primeira fixação, duração total das fixações e probabilidade de retornos ao estímulo) trouxeram informações complementares à aplicação tradicional da tarefa de leitura de palavras e pseudopalavras e que podem ser relevantes para um diagnóstico clínico mais preciso, bem como para desenhar intervenções para pacientes com dislexia. Também é importante ressaltar que as dislexias podem ter manifestações leves, moderadas e graves. Quando a dificuldade é muito leve, por exemplo, em uma fase muito inicial de quadros neurodegenerativos, instrumentos que possam detectar comportamentos que sugerem uma disfunção incipiente podem ser relevantes para a prática clínica. Como perspectiva futura, são necessários trabalhos com amostras maiores e mais representativas da população, ou seja, com mais variabilidade em termos de escolaridade, idade, nível socioeconômico e região. A partir disso, também será necessário criar ferramentas que facilitem a obtenção, análise e interpretação das informações que os dados obtidos por rastreamento ocular podem trazer, para que os profissionais que atuam no diagnóstico e intervenção em pacientes com dislexia possam se beneficiar dessa ferramenta em seu dia-a-dia e em nível individual. Há um caminho longo a percorrer nesse sentido e este trabalho visa inspirar a ampliação de estudos nesta área.

## CONCLUSÃO

Testes de leitura de palavras e pseudopalavras são frequentemente empregados para investigar quadros de dislexia do desenvolvimento e adquiridas, avaliando a preservação e funcionamento das rotas de leitura. O rastreador ocular é um instrumento que analisa o desempenho na leitura de forma objetiva, podendo ser uma ferramenta adicional para investigar esses aspectos, porém ainda não é utilizado na prática clínica no Brasil. O objetivo do presente estudo, de investigar os padrões oculomotores durante a leitura em Português em uma população cognitivamente saudável, foi contribuir para a caracterização do perfil típico em leitores do português- brasileiro, mostrando sobretudo os efeitos de lexicalidade, de frequência e extensão. Este trabalho é uma prova de conceito de como o rastreador ocular pode auxiliar na obtenção de dados e valores de referência para fins de pesquisa e aplicabilidade clínica na investigação diagnóstica, onde déficits sutis podem vir a ser detectados e algumas características do comportamento oculomotor podem estar mais associadas com bom desempenho na leitura. Para isso, outros estudos são sugeridos em língua portuguesa considerando amostras maiores, diferentes populações, outros estímulos linguísticos, como frases e textos, e investigação da correlação do comportamento oculomotor com funções cognitivas superiores, sobretudo com a memória operacional fonológica e a fluência verbal.
